# Negative effect of cyclin D1 overexpression on recurrence-free survival in stage II-IIIA lung adenocarcinoma and its expression modulation by vorinostat in vitro

**DOI:** 10.1186/s12885-015-2001-7

**Published:** 2015-12-17

**Authors:** Eunju Lee, DongHao Jin, Bo Bin Lee, Yujin Kim, Joungho Han, Young Mog Shim, Duk-Hwan Kim

**Affiliations:** 1 Department of Molecular Cell Biology, Sungkyunkwan University School of Medicine, #300 Chunchun-dong, Jangan-Ku, Kyunggido Suwon, 440-746 Korea; 2 Department of Pathology, Samsung Medical Center, #50 Ilwon-dong, Kangnam-Ku, Seoul 135-710 Korea; 3 Department of Thoracic and Cardiovascular Surgery, Samsung Medical Center, #50 Ilwon-dong, Kangnam-Ku, Seoul 135-710 Korea; 4 Center for Genome Research, Samsung Biomedical Research Institute, Rm B155, #50 Ilwon-dong, Kangnam-Ku, Seoul 135-710 Korea

**Keywords:** Lung cancer, Vorinostat, Cyclin D1, Histone modification, Survival

## Abstract

**Background:**

This study was aimed at identifying prognostic biomarkers for stage II-IIIA non-small cell lung cancer (NSCLC) according to histology and at investigating the effect of vorinostat on the expression of these biomarkers.

**Methods:**

Expression levels of cyclin D1, cyclin A2, cyclin E, and p16 proteins that are involved in the G_1_-to-S phase progression of cell cycle were analyzed using immunohistochemistry in formalin-fixed paraffin-embedded tissues from 372 samples of stage II-IIIA NSCLC. The effect of vorinostat on the expression of these proteins, impacts on cell cycle, and histone modification was explored in lung cancer cells.

**Results:**

Abnormal expression of cyclin A2, cyclin D1, cyclin E, and p16 was found in 66, 47, 34, and 51 % of 372 cases, respectively. Amongst the four proteins, only cyclin D1 overexpression was significantly associated with poor recurrence-free survival (adjusted hazard ratio = 1.87; 95 % confidence interval = 1.12 – 2.69, *P* = 0.02) in adenocarcinoma but not in squamous cell carcinoma (*P* = 0.44). Vorinostat inhibited cell cycle progression to the S-phase and induced down-regulation of cyclin D1 in vitro. The down-regulation of cyclin D1 by vorinostat was comparable to a siRNA-mediated knockdown of cyclin D1 in A549 cells, but vorinostat in the presence of benzo[a]pyrene showed a differential effect in different lung cancer cell lines. Cyclin D1 down-regulation by vorinostat was associated with the accumulation of dimethyl-H3K9 at the promoter of the gene.

**Conclusions:**

The present study suggests that cyclin D1 may be an independent prognostic factor for recurrence-free survival in stage II-IIIA adenocarcinoma of lung and its expression may be modulated by vorinostat.

**Electronic supplementary material:**

The online version of this article (doi:10.1186/s12885-015-2001-7) contains supplementary material, which is available to authorized users.

## Background

Lung cancer is the leading cause of cancer-related deaths worldwide and despite significant advances in the diagnosis and treatment of the disease, the current 5-year survival rate remains low at 15 %. The poor prognosis is partially due to the high rate of recurrence after surgery, where the recurrence rate is as high as 20–40 % even for a stage I non-small cell lung cancer (NSCLC) [1, 2]. The recurrence is the result of local and distant metastasis of residual cancer cells after surgery. A number of studies have been conducted to identify specific adjuvant therapy in order to eliminate occult micro-metastases after curative surgical resection and improve survival. Adjuvant chemotherapy is recommended for some patients with resected stage II-IIIA NSCLC but controversy continues regarding its need for stage I NSCLC. The role of adjuvant chemotherapy in patients with stage IB NSCLC is not well established, and it is recommended only for certain patient cases [3].

In the last 10 years, adjuvant chemotherapy for patients with completely resected stage II-IIIA NSCLC has usually employed platinum-based chemotherapy. After a history of negative trials over the last few decades, some progress has been made in overall survival after platinum-based chemotherapy. Two recent meta-analysis of randomized controlled trials showed an absolute 5-year survival benefit of 5 to 10 % irrespective of the associated drugs such as vinorelbine or etoposide, with the main survival advantage being in the patients with stage II-IIIA NSCLC [4, 5]. With a better understanding of the biology of lung cancer in recent years, several groups have proposed novel strategies targeting the epidermal growth factor receptor (EGFR), other receptor and non-receptor tyrosine kinases, and vascular endothelial growth factor (VEGF) pathways [3].

A balance between stimulators and inhibitors of cell proliferation tightly regulates the cell cycle and a disorganization of the cell cycle leads to an uncontrolled cellular proliferation of residual cancer cells after curative resection. Chemotherapeutic agents that target and disrupt different phases of the cell cycle have been developed over the past few years. Among them, histone deacetylase inhibitors (HDACIs) modify the acetylation state of histone tails and induce cell cycle arrest at both G_1_ and G_2_ phases. Vorinostat, also known as suberoylanilide hydroxamic acid (SAHA), was the first HDACI to be approved by the United States Food and Drug Administration (FDA) for treatment of refractory cutaneous T-cell lymphoma [6]. Vorinostat also causes cell growth inhibition, differentiation, and apoptosis of lung cancer cells in vitro through various mechanisms [7–10].

To understand the expression pattern and prognostic significance of key proteins involved in the G_1_-to-S phase progression of the cell cycle in stage II-IIIA NSCLC and to investigate whether vorinostat can modulate expression of these proteins, we analyzed the expression patterns of cyclin A2, cyclin D1, cyclin E, and p16 proteins in formalin-fixed paraffin-embedded tissues from 372 patients with stage II-IIIA NSCLC and assessed the effect of vorinostat on their expression in lung cancer cells. A serious problem in the treatment of lung cancer is that some patients continue to smoke even after a lung cancer diagnosis. The continuous exposure to tobacco smoke may influence the effect of chemotherapeutic agents [11]. Therefore, we carried out the in vitro study with and without exposure to benzo[a]pyrene (B[a]P).

## Results

### Expression patterns of the four proteins

**Fig. 1:**
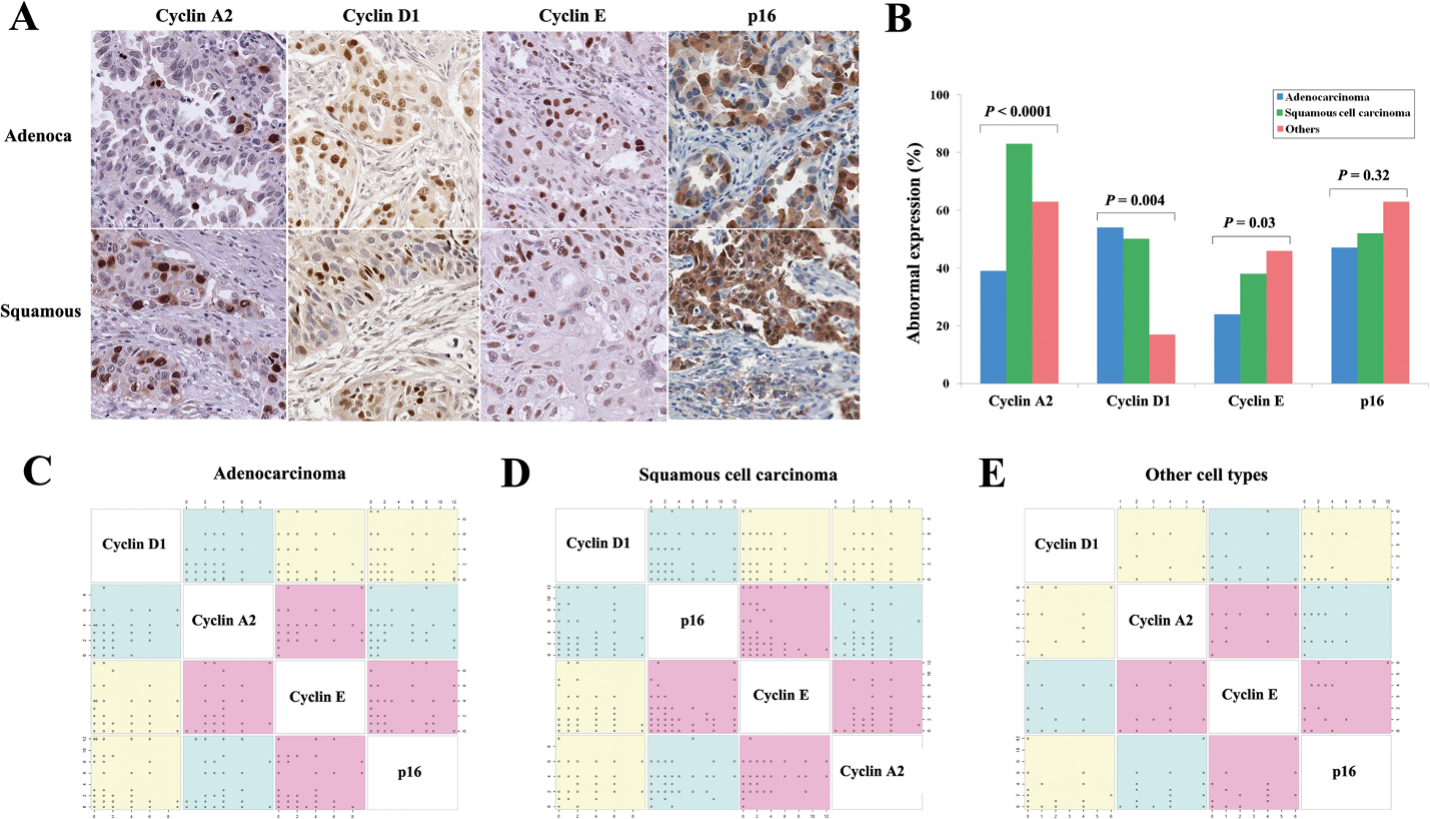
Expression patterns of four proteins according to histology. **a** Immunohistochemical staining of 4 proteins was performed in 372 formalin-fixed paraffin-embedded tissues. Representative positive staining is shown in adenocarcinoma (upper) and squamous cell carcinoma (lower). (Magnification ×200). **b** Prevalence of abnormal expression of 4 proteins was compared according to histology. *P*-values were based on Pearson’s chi-square or Fisher’s exact test. **c**-**e** Correlations among expression levels of 4 proteins were analyzed using Spearman’s rank correlation coefficient in adenocarcinoma (**c**), squamous cell carcinoma (**d**), and other cell types (**e**). The numbers on either side of plots indicate composite scores that were calculated by multiplying the intensity score and the proportion score of positive staining tumor cells. Violet color indicates a significant Spearman’s correlation between two proteins (*P* < 0.05)

A total of 372 patients with stage II-IIIA NSCLC participated in this study. The clinicopathological characteristics according to histology are described in Table 1. Representative examples of nuclear immunostaining for the four proteins are shown in Fig. 1a. A composite score for each staining was calculated by multiplying the intensity and percentage scores. Abnormal expression was defined when a composite score was greater than or equal to two for cyclin A2, cyclin D1, and cyclin E and less than two for p16. Abnormal expression was detected in 66 % of 372 patients for cyclin A2, 47 % for cyclin D1, 34 % for cyclin E, and 51 % for p16. Abnormal expression of these four proteins was compared according to histology (Fig. 1b): a low prevalence of the overexpression of cyclin A2 (*P* < 0.0001) and cyclin E (*P* = 0.003) was shown in lung adenocarcinoma as compared to other cell types. As protein-protein interactions play a fundamental role in many biological processes, a correlation analysis of the expression of these four proteins was performed according to histology. The expression levels of cyclin E correlated with those of cyclin A2 (Spearman’s rank correlation coefficient [*r*_s_] = 0.38, *P* < 0.0001) and p16 (*r*_s_ = 0.32, *P* = 0.0003) in adenocarcinoma (Fig. 1c). This was also similar to patterns in squamous cell carcinoma (Fig. 1d) and other cell types (Fig. 1e). However, no correlation was found between cyclin D1 and other proteins.

**Table 1:** Clinicopathological characteristics (*N* = 372)

Variables	Adenoca (*N* = 140)	Squamous (*N* = 201)	Others (*N* = 31)	*P*-value
Age^a^	59 ± 11	62 ± 8	59 ± 10	0.03
Tumor size (cm)^a^	4.5 ± 2.7	5.3 ± 2.4	6.3 ± 2.8	<0.0001
Pack-years (smoking)^a^	19 ± 24	37 ± 19	40 ± 35	<0.0001
Sex				
Male	87	192	27	
Female	53	9	4	<0.0001^c^
Smoking status				
Never	72	14	7	
Former	29	62	5	
Current	39	125	19	<0.0001
Pathologic stage				
IIA	44	71	10	
IIB	46	54	13	
IIIA	50	76	8	0.42
Differentiation^b^				
Well	25	27	1	
Moderate	47	120	1	
Poorly	42	40	2	
Undifferentiated	2	2	3	<0.0001^c^
Adjuvant chemotherapy				
No	115	163	26	
Yes	25	38	5	0.92
Adjuvant radiotherapy				
No	88	139	22	
Yes	52	62	9	0.42
Recurrence				
No	35	114	16	
Yes	105	87	15	<0.0001

*Abbreviations*: *Adenoca*, adenocarcinoma; *Squamous*, squamous cell carcinoma

^a^Values indicate mean ± standard deviation

^b^Differentiation data are missing for 60 patients

^c^Fisher’s exact test

### Survival analysis

**Fig. 2:**
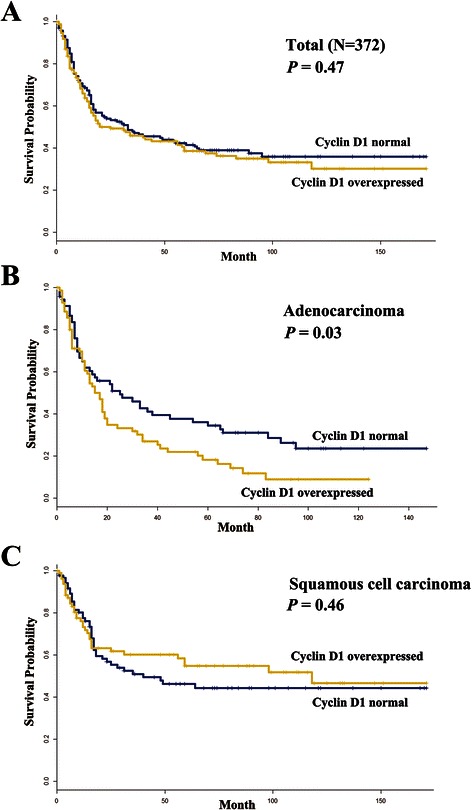
Kaplan-Meier survival curves in stage II-IIIA NSCLC. Recurrence-free survival was compared according to expression status of cyclin D1 in 372 patients (**a**), 140 patients with adenocarcinomas (**b**), and 201 patients with squamous cell carcinomas. *P*–values were based on the log-rank test

The effect of the abnormal expression of individual proteins on recurrence-free survival (RFS) and overall survival was analyzed in 372 NSCLCs. Abnormal expression of cyclin A2, cyclin D1, cyclin E, and p16 was not associated with overall survival (data not shown). In addition, no association was found between RFS and the abnormal expression of cyclin A2, cyclin E, and p16 (Additional file 1: Figure S1). However, cyclin D1 overexpression was found to be significantly associated with poor RFS in adenocarcinoma (*P* = 0.03; Fig. 2b), not in squamous cell carcinoma (*P* = 0.46; Fig. 2c). The median duration of RFS was 15 months and 25 months for adenocarcinoma patients with and without cyclin D1 overexpression, respectively. Cox proportional hazards analysis showed that adenocarcinoma patients with cyclin D1 overexpression were found to have a 1.87 (95 % confidence interval = 1.12 – 2.69, *P* = 0.02) times poorer RFS than those without (Table 2), after adjusting for age, tumor size, pathologic stage, and cyclin E expression. The cyclin A2, cyclin E, and p16 proteins were entered into the multivariate analysis one by one due to collinearity, but their expression was not associated with RFS (data not shown).

**Table 2:** Cox proportional hazards analysis^a^ for recurrence-free survival

Histology	Cyclin D1 overexpression	HR	95 % CI	*P*-value
Total	No	1.00		
(*N* = 372)	Yes	1.10	0.83–1.46	0.51
Adenocarcinoma	No	1.00		
(*N* = 140)	Yes	1.87	1.12–2.69	0.02
Squamous cell ca	No	1.00		
(*N* = 201)	Yes	0.84	0.55–1.29	0.44

*Abbreviations*: *HR* hazard ratio; *CI* confidence interval; *Squamous cell ca*, squamous cell carcinoma

^a^adjusted for age, tumor size, pathologic stage, and cyclin E expression

### Vorinostat inhibits cell growth

**Fig. 3:**
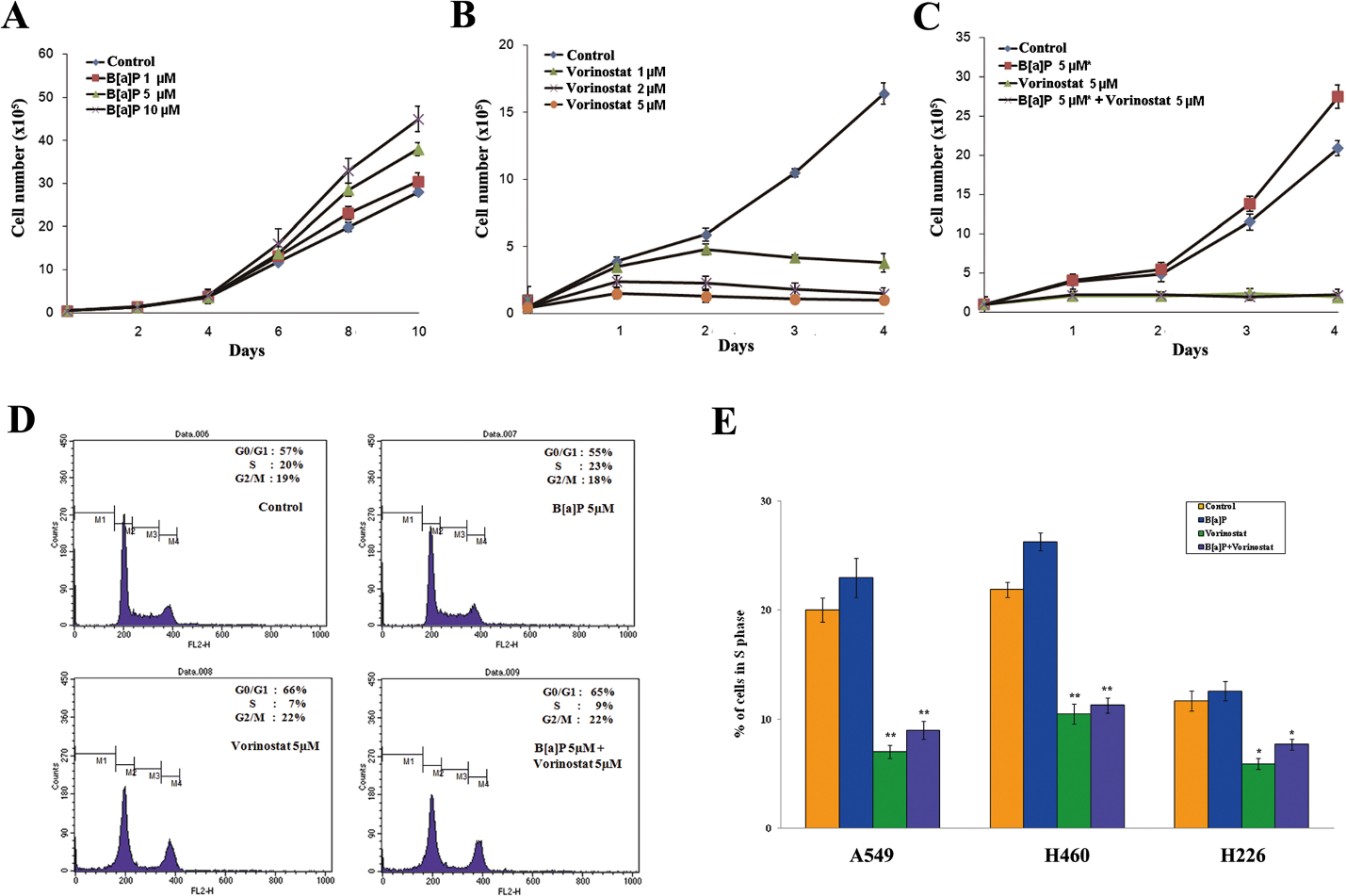
The effect of vorinostat on cell growth and cell cycle in vitro. **a** & **b** A549 cells were cultured with B[a]P or vorinostat at the concentrations indicated and for the times indicated to analyze their effect on cell growth. **c** To study the effect of vorinostat on cell growth in A549 cells exposed to B[a]P as long as possible, A549 cells were first pretreated with 5 μM B[a]P for 9 days (asterisk), and then followed by combination with 5 μM vorinostat for the times indicated. Viable cells were counted using trypan blue at each experiment, and data are presented as the mean ± standard error (SE) of triplicate experiments. **d** A549 cells were cultured with 5 μM vorinostat and/or 5 μM B[a]P as described in the Materials and Methods. After incubation, the cells were stained with propidium iodide, and cell cycle distributions were analyzed by flow cytometry. **e** The effect of vorinostat and/or B[a]P on cell cycle was also analyzed in H460 and H226 lung cancer cell lines in triplicate. The Y-axis indicates the percentage of cells in the S phase of cell cycle, and error bars indicate one standard deviation. The percentage of cells in the S phase was compared between vorinostat-treated and control cells and between B[a]P-treated and B[a]P/vorinostat-treated cells. The difference was analyzed using paired Student t-test. The symbols * and ** denote significant differences at *P* < 0.05 and *P* < 0.01, respectively

In our clinical samples, cyclin D1 overexpression was significantly associated with RFS in stage II-IIIA adenocarcinoma. We used A549, a human lung adenocarcinoma epithelial cell line that expresses relatively high levels of cyclin D1, as our model to analyze the effect of vorinostat on cell growth. B[a]P increased cell proliferation, while vorinostat significantly decreased proliferation in a time- and dose-dependent manner (Fig. 3a and b). In order to examine the effect of vorinostat on cell growth in cells exposed to B[a]P as long as possible, we pretreated A549 cells with 5 μM B[a]P for 9 days and incubated the cells with 5 μM vorinostat in combination for another 4 days (Fig. 3c). Cell proliferation in the cells exposed to B[a]P was also reduced by vorinostat, which showed the same pattern as the inhibition of cell proliferation by vorinostat in the absence of B[a]P.

### Vorinostat induces G_1_-S arrest in lung cancer cells

Cell cycle was evaluated using flow cytometry in A549, H460, and H226 cells treated with 5 μM B[a]P and/or 5 μM vorinostat: vorinostat did have a substantial effect on G_1_-S cell cycle arrest. The proportion of S phase cells in the cell lines substantially decreased as compared to the control by treatment with 5 μM vorinostat for 1 day. The proportion of S phase cells in A549 cells decreased from 20 to 7 % by vorinostat (Fig. 3d). The proportion of S phase cells in A549 cells exposed to 5 μM B[a]P decreased from 23 to 9 % by 5 μM vorinostat (*P* < 0.05; paired t-test). Vorinostat also blocked cell cycle progression to the S phase in H460 (large cell carcinoma cell line) and H226 (squamous cell carcinoma cell line) cells irrespective of exposure to B[a]P (Fig. 3e). These observations suggest that the effect of vorinostat on G_1_-S arrest of the cell cycle may not be cell type-specific in lung cancer.

### The effect of vorinostat on cyclin D1 expression is comparable to cyclin D1 siRNA

**Fig. 4:**
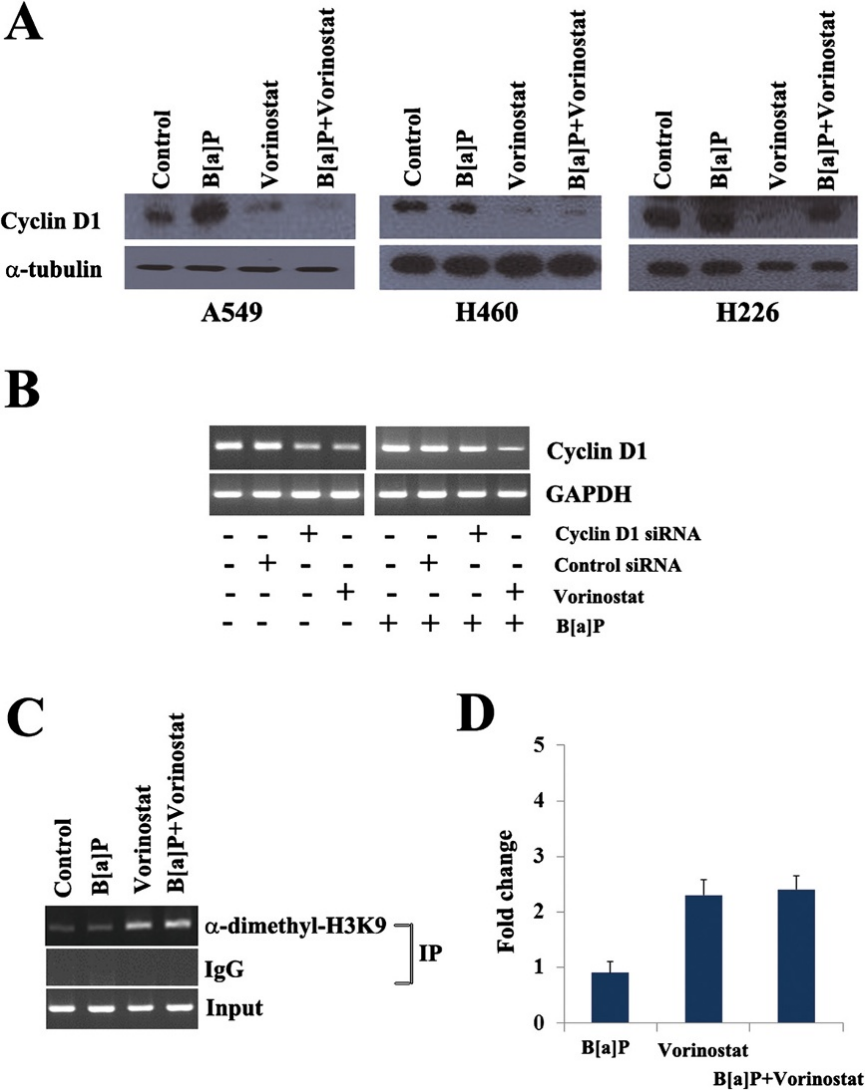
The effect of vorinostat on cyclin D1 expression and histone modification. **a** Cyclin D1 expression was analyzed in A549, H460, and H226 cells treated with vorinostat and/or B[a]P. Cellular lysate protein (30 μg/lane) was loaded onto a 10 % SDS/PAGE gel, electrophoresed, and subsequently immunoblotted with a cyclin D1 antibody. **b** The effect of vorinostat and cyclin D1 siRNA on cyclin D1 down-regulation was compared in A549 cells. The cells were transfected with 75 nM of cyclin D1 siRNA for 2 days (lanes 2 & 5) or with 5 μM vorinostat for 1 day (lanes 3 & 6), The cyclin D1 expression in the presence of B[a]P was analyzed after pretreatment of cells with B[a]P for 9 days (lane 4 through lane 6). **c** & **d** Dimethylation of H3K9 histone tail at the promoter of cyclin D1 was analyzed using chromatin immunoprecipitation (ChIP) after incubating A549 cells as described in the Materials and Methods. The intensities of bands were measured using a densitometer and are presented as relative band intensities compared to control. Error bars indicate one standard deviation

The effect of vorinostat on cyclin D1 expression was further analyzed because of our finding that cyclin D1 was significantly associated with poor RFS in stage II-IIIA lung adenocarcinoma. Cyclin D1 was found to be down-regulated in response to vorinostat in A549, H460, and H226 cells, but the effect varied according to the cell lines in presence of B[a]P (Fig. 4a): cyclin D1 down-regulation by vorinostat was minimal in H226 cells exposed to B[a]P. To understand if the effect of vorinostat on cyclin D1 down-regulation was comparable to a cyclin D1 knockdown, we treated A549 cells either with vorinostat or cyclin D1 siRNA in absence or presence of B[a]P (Fig. 4b). In absence of B[a]P, cyclin D1 siRNA and vorinostat showed similar effects on cyclin D1 down-regulation (lanes 3 and 4, respectively). However, siRNA-mediated knockdown of cyclin D1 (lane 7) was not as effective as vorinostat (lane 8) in A549 cells exposed to B[a]P. Based on this observation, cyclin D1 may be one of the targets of vorinostat in lung adenocarcinoma cells irrespective of exposure to B[a]P.

### Vorinostat induces histone modification at the promoter of cyclin D1

To understand the mechanism underlying the cyclin D1 down-regulation in response to vorinostat, we analyzed the modification of H3K9 histone tail at the promoter of cyclin D1 using chromatin immune-precipitation (ChIP) in A549 cells. Cyclin D1 promoter contains multiple transcription factor binding sites, including AP-1, NF-қB, E2F, and Oct-1 (reviewed in ref. [12]). ERK pathway induces cyclin D1 promoter activity through Ets or AP-1 response elements at cyclin D1 promoter, and benzo[a]pyrene-induced cell cycle progression occurs through the ERKs/cyclin D1 pathway [13]. Therefore, we analyzed histone modifications at the AP-1 response element region at the cyclin D1 promoter. The level of dimethyl-H3K9 at the promoter of cyclin D1 was increased in cells treated with vorinostat alone as well as with vorinostat in combination with B[a]P as compared to the control (Fig. 4c and d): the quantitative analyses revealed that vorinostat treatment in A549 cells increased the levels of dimethyl-H3K9 at the cyclin D1 promoter 2.3 times compared to the control (*P* = 0.04, Wilcoxon-rank sum test). These observations suggest that cyclin D1 down-regulation may be associated with the accumulation of dimethyl-H3K9 at the promoter of cyclin D1.

## Discussion

Given that adjuvant chemotherapy is effective in some patients with stage II-IIIA NSCLC, discovery of novel chemotherapeutic agents has become increasingly important in improving patient survival. Standard adjuvant chemotherapy in resected NSCLC is usually based on the use of a cytotoxic agent such as cisplatin. Cisplatin binds to DNA and forms a spectrum of intra- and inter-strand DNA crosslinks, and the resulting cisplatin-DNA adduct interferes with DNA replication. The benefits of platinum-based adjuvant therapy remain modest, with improvements in 5-year survival of 5-10 %, and there continues to be a need for development of novel adjuvant chemotherapeutic agents. Recently, biomarker-based adjuvant chemotherapy has been reported as a model of novel targeted therapy to further improve patient survival after surgery. In this study, the effect of vorinostat in combination with B[a]P on cell proliferation was tested with the same concentrations (5 μM) for two agents. And, low dose (1 μM) treatment of vorinostat in A549 cells exposed to B[a]P in vitro also inhibited cell proliferation to a similar degree (Additional file 2: Figure S2): the number of viable cells significantly decreased at 72 h (*P* = 0.01) and 96 h (*P* = 0.008, Student t-test) after treatment with vorinostat compared to the control. Vorinostat also significantly decreased the number of viable cells in A549 cells exposed to B[a]P (*P* = 0.009 at 72 h and *P* = 0.003 at 96 h; Student t-test).

We evaluated vorinostat as a therapeutic candidate against cyclin D1 in lung cancer cells because vorinostat is known to suppress cyclin D1 expression in mantle cell lymphoma cells [14], colon cancer cells [15], renal cancer cells [16], and JB6 mouse epidermal Cl 41 cells [17] and because cyclin D1 overexpression was significantly associated with poor RFS in stage II-IIIA lung adenocarcinoma (Fig. 2). Vorinostat suppressed cyclin D1 expression irrespective of exposure to B[a]P in A549 cells. This effect, however, was different in several other lung cancer cell lines tested according to the presence of B[a]P (Fig. 4a). Cyclin D1 plays an essential role in the oncogenic transformation and its overexpression occurs in approximately 50 % of NSCLC with demonstrated tumorigenesis seen with human bronchial epithelial cells exposed to B[a]P. The prognostic significance of cyclin D1 in NSCLC has been reported by a number of groups, with conflicting results. Some studies reported cyclin D1 overexpression as an independent negative or some a positive prognostic indicator, whereas others failed to find a significant association of cyclin D1 overexpression with prognosis [18]. Recently, Zhang et al. [19] performed a meta-analysis of reported 24 studies with 2731 NSCLC patients to understand the prognostic significance of cyclin D1 overexpression in NSCLC and found that cyclin D1 overexpression was not associated with overall survival in NSCLC. We also did not find an association between cyclin D1 overexpression and overall survival in NSCLC (data not shown). The overexpression of cyclin E is common (~45 %) in NSCLC, but the impact of its overexpression on survival remains unclear. The overexpression of cyclin A is known to be associated with poor overall survival in NCSLC and p16 is inactivated in approximately 50 % of NSCLC cases, but there is no convincing evidence to suggest that p16 is a significant prognostic marker in NSCLC. Only cyclin D1 overexpression in the present study was significantly associated with poor RFS in stage II-IIIA lung adenocarcinoma, suggesting that histology may function as an effect modifier in the relationship between RFS and protein expression.

Vorinostat leads to cyclin D1 suppression in vitro and this could be via different mechanisms. For example, vorinostat suppresses cyclin D1 in mantle cell lymphoma cells by blocking the translation of cyclin D1 via inhibiting the phosphatidylinositol 3-kinase (PI3K)/Akt/mTOR/eIF4E-BP pathway most likely by PI3K inhibition [14]. Vorinostat also down-regulates cyclin D1 expression by decreasing histone deacetylase activity [15] or by reducing cyclin D1 mRNA stability [16]. To understand molecular mechanisms underlying cyclin D1 down-regulation by vorinostat in A549 cells, we analyzed the modification of H3K9 in response to vorinostat using chromatin immunoprecipitation. Chromatin structure is dynamically altered by reversible modifications of core histones through the activities of histone acetyltransferases (HATs) and HDACs, and acetylation of core histones is linked to transcriptional activation. B[a]P is known to induce histone modification in human cancer cells [20, 21]. Vorinostat is also known to increase acetylation of histones at the promoter of p21^WAF1^ in bladder carcinoma cells [22], multiple myeloma cells [23], and endometrial cancer cells [24]. In this study, vorinostat increased the levels of dimethyl-H3K9 at the promoter region of cyclin D1 in A549 cells exposed to B[a]P, suggesting that vorinostat may down-regulate cyclin D1 through the modification of the chromatin structure at the promoter of the gene.

B[a]P is the carcinogenic component of polycyclic aromatic hydrocarbons, one of the main carcinogens in cigarette smoke, and is regarded as a mediator of lung cancer. B[a]P is metabolized by cytochrome P450 enzyme to benzo[a]pyrene-7,8-diol-9,10-epoxide (B[a]PDE), which is highly cytotoxic, mutagenic, and carcinogenic [25, 26]. B[a]PDE enhances cyclin D1 expression in bronchial epithelial cells, and the increased cyclin D1 promotes malignant transformation of the cells. The PI3K/Akt pathway, as well as downstream MAPK and p70S6 kinase, are known to be involved in B[a]PDE-induced cyclin D1 expression [13]. B[a]P exposure in human embryo lung fibroblasts accelerates the G_1_-S transition by activating MAPK and inducing cyclin D. Treatment with antisense cyclin D1 or antisense CDK4 completely inhibited B[a]P-induced cell cycle progression at the G_1_-S checkpoint [27]. In the present study, B[a]P increased the expression of cyclin D1 in A549 and H226 cells but not in H460 cells (Fig. 4a). The lack of up-regulation of cyclin D1 in H460 cells treated with B[a]P may result from the presence of mutant K-ras in these cells.

In this study, vorinostat also repressed the expression of cyclin E, cyclin A2, and cyclin B1 in A549, H460, and H226 cells (Additional file 3: Figure S3). But, these proteins were not further evaluated because altered expression of the proteins was not associated with patient prognosis in stage II-IIIA NSCLC (Additional file 1: Figure S1). Our study was severely limited by several factors. First, the effect of vorinostat needs to be evaluated prospectively in patients with stage II-IIIA NSCLC in which cyclin D1 is overexpressed. Second, vorinostat has known toxicity and significant side effects as with most chemotherapeutic agents. To minimize the side effects of vorinostat and maximize its therapeutic effect, a combination therapy of vorinostat with other drugs such as a DNMT inhibitor, proteasome inhibitor, or anti-Trail antibody also needs to be considered. Third, cyclin D1 can also be repressed through other mechanisms such as autophagy and senescence after vorinostat [28]. Further study is required to investigate possible mechanisms related to cyclin D1 down-regulation in response to vorinostat and B[a]P.

## Conclusions

In summary, the present study suggests that cyclin D1 overexpression may be significantly associated with poor RFS in stage II-IIIA lung adenocarcinoma and its expression be modulated by vorinostat. We recommend vorinostat as an adjuvant chemotherapeutic agent for patients with stage II-IIIA adenocarcinoma in which cyclin D1 is overexpressed.

## Methods

### Ethics statement

This retrospective study was approved by the Samsung Medical Center (SMC) Institutional Review Board (IRB # : 2010-07-204). All patients’ records and information were anonymized and de-identified prior to analysis.

### Study population

A total of 372 stage II-IIIA NSCLC patients who received curative surgical resection between September 1994 and December 2004 at the Samsung Medical Center in Seoul, Korea, participated in this study. Tumor samples were obtained in accordance with the Declaration of Helsinki. A written informed consent for the use of formalin-fixed, paraffin-embedded tissues was obtained from all of the patients before surgery. Information on patient demographics was obtained from an interviewer-administered questionnaire, and post-operative follow-up for detection of death or recurrence, which was evaluated as of August 31, 2014, was conducted as previously described [2]. Median duration of follow-up after surgery was 32 months. Lung cancer staging was determined according to the guidelines of the American Joint Committee on Cancer (AJCC) TNM staging system [29].

### Cell culture

A549, H460, and H226 lung cancer cell lines were obtained from the American Type Culture Collection (Manassas, VA), and the cells were cultured in regular RPMI-1640 medium (Lonza, Walkerville, MD) supplemented with 10 % fetal bovine serum (Hyclone, Logan, UT), 1.0 mM of sodium pyruvate (Sigma-Aldrich, St. Louis, MO) and 1 % HEPES buffer at 37 °C with 5 % CO_2_. The cells were tested and authenticated using real-time PCR and capillary sequencing in February 2014.

### In vitro growth assay

A549 cells were seeded in six-well plates with 2.0 × 10^5^ in each well. They were treated with vorinostat alone or in combination with B[a]P. For analysis of dose- and time-dependent effects of vorinostat and B[a]P on cell growth, A549 cells were incubated at different concentrations of B[a]P (0, 1, 5, 10 μM) for 10 days, and vorinostat (0, 1, 2, 5 μM) for 4 days. In addition, to analyze the effect of vorinostat on cell proliferation in cells exposed to B[a]P, A549 cells were pretreated with 5 μM B[a]P for 9 days and followed by a combination with 5 μM vorinostat for another 1 day. A number of viable cells were counted using trypan blue or the Vybrant MTT Cell Proliferation Assay Kit (Life Technologies) according to the manufacturer’s instructions. The experiments were performed in triplicate.

### Flow cytometry analysis of cell cycle

For cell cycle analysis, the cells were cultured in six-well plates to 70–80 % confluence with culture medium, and subsequently treated with 5 μM B[a]P for 10 days, or 5 μM vorinostat for 1 day, or 5 μM B[a]P for 9 days followed by co-incubation with 5 μM vorinostat for an additional day. After incubation of A549, H460, and H226 cells with 5 μM vorinostat and/or 5 μM B[a]P, the cells were harvested and washed in PBS, and then fixed in 70 % ice-cold ethanol for 24 h. The fixed cells were stained with propidium iodide (50 mg/ml) containing 5 mg/ml RNase A (Boehringer Ingelheim, Ingelheim, Germany) and 0.1 % Triton X-100 and incubated for 15 min in dark. Fluorescence for cell cycle distribution was detected by the FACScan flow cytometer (Becton-Dickson, San Jose, CA) and resulting data was analyzed using the ModFit LT version 3.0 (Verity Software House, Topsham, ME). At least 10,000 cells were examined for each sample and the experiments were repeated at least three times.

### Western blot analysis

Cyclin D1 expression in response to B[a]P and vorinostat was analyzed by immune-blotting using antibodies against cyclin D1 (DCS6, #2926, Cell Signaling, Danvers, MA) and α-tubulin (T6074, Sigma-Aldrich, St. Louis, MO) as a loading control according to standard procedures.

### Reverse transcription polymerase chain reaction (RT-PCR)

Cells were harvested and washed in ice-cold PBS after culture. Total RNA was isolated using the RNeasy Mini Kit (Qiagen, Valencia, CA, USA) according to the manufacturer’s instructions. RT-PCR was carried out in a tube containing 0.5 μg of total RNA and primers specific to cyclin D1 at a final concentration of 0.5 μM using the OneStep RT-PCR kit (Qiagen) according to the manufacturer’s protocols. The sequences of primers used for RT-PCR were as follows: cyclin D1 forward primer 5′-TGTTCGTGGCCTCTAAGATGA-3′, reverse primer 5′-GCTTGACTCCAGAAGGGCTT-3′; GAPDH forward primer 5′- AACTTTGGCATTGTGGAAGG, reverse primer 5′-TGTGAGGGAGATGCTCAGTG.

### Cyclin D1 knockdown using small interfering RNA (siRNA)

To evaluate if the effect of vorinostat on cyclin D1 down-regulation was comparable to a cyclin D1 knockdown in lung cancer cells, A549 cells (2.0 × 10^5^ cells per well) were seeded in six-well plates, and at 70 %–80 % confluence the cells were treated with 5 μM vorinostat for 1 day or with 75 nM of cyclin D1 siRNA (SI 02654547, Qiagen) for 2 days. A549 cells were also pretreated with B[a]P for 9 days and then treated with cyclin D1 siRNA and/or vorinostat in the same way that was performed in the absence of B[a]P. Transfection of cyclin D1 siRNA was performed using FlexiTube siRNA & HiPerFect Transfection Reagent (Qiagen GmbH, Hilden, Germany) according to the manufacturer’s protocol.

### Chromatin immunoprecipitation

For the analysis of dimethylation of the H3K9 histone tail at the promoter of cyclin D1, A549 cells were cultured with 5 μM B[a]P for 10 days, or 5 μM vorinostat for 1 day, or 5 μM B[a]P for 9 days followed by co-incubation with 5 μM vorinostat for an additional 24 h. After 48 h of culture, chromatin immunoprecipitation (ChIP) was performed using a ChIP assay kit (Upstate Biotechnology, Lake Placid, NY) according to the manufacturer’s protocols. The antibodies used for ChIP were anti-dimethyl-histone H3K9 (lys9) and normal IgG (negative control) (Upstate Biotechnology). The primer sequences for promoter amplification of cyclin D1 were 5′- CTGCCTTCCTACCTTGACCA -3′ (forward) and 5′- GAAGGGACGTCTACACCCC -3′ (reverse).

### Immunohistochemistry

Tissue microarrays (TMAs) of NSCLC and immunohistochemical staining of cyclin A2, cyclin D1, cyclin E, and p16 were conducted as previously described [30]. The antibodies used were as follows: cyclin A2 (BF683, Cell Signaling, Danvers, MA, USA), cyclin D1 (DCS6, Cell Signaling), cyclin E (HE12, Cell signaling), and p16 (Ab-16, Neomarker, Fremont, CA). All available slides were reviewed by two authors (EY Cho, D-H Kim), who were blinded to all clinicopathological variables, to reduce inter-observer variability. The expression of individual proteins was assigned to the extent and intensity of tumor cells that stained positively in nucleus. Cytoplasmic reactivity, if present, was disregarded. The expression levels of the four proteins were calculated by multiplying the intensity score (0, none; 1, weak; 2, moderate; 3, strong) with the proportion score of positive staining tumor cells (0, absent; 1, 0–10 %; 2, 10–50 %; 3, 50–80 %; 4, > 80 %).

### Statistical analysis

In the univariate analysis, Pearson’s chi-square test (or Fisher’s exact test) and t-test (or Wilcoxon rank-sum test) were used for analysis of categorical and continuous variables, respectively. Correlations among expression levels of the four proteins were compared using Spearman’s rank correlation coefficients. The effect of protein expression on recurrence-free survival (RFS) or overall survival was analyzed by Kaplan-Meier survival curves, and the significance of differences in survival between the two groups was evaluated by the log-rank test. Cox proportional hazards regression model was used to estimate adjusted hazard ratios with 95 % confidence interval for independent predictor variables. All statistical analyses were two-sided, with a 5 % type I error rate.

## Additional files

Additional file 1: Figure S1. Recurrence-free survival in stage II-IIIA NSCLC. Recurrence-free survival was compared according to expression statuses of cyclin A2, cyclin E, and p16. Data were stratified according to histology. The yellow and blue lines indicate groups with and without abnormal expression of each protein, respectively. *P*–values were based on the log-rank test. (TIFF 2996 kb)
Additional file 2: Figure S2. Effect of low dose vorinostat on cell proliferation. A549 cells were pre-treated with 5 μM B[a]P for 9 days and then incubated in combination with 1 μM vorinostat for the indicated hours. The total number of viable cells at each time point was determined by MTT assay. The y-axis indicates cell numbers relative to time zero. Each value represents mean ± standard deviation of three experiments. (TIFF 1042 kb)
Additional file 3: Figure S3. The effect of vorinostat on expression of cyclins. The expression levels of cyclin E, cyclin A2, and cyclin B1 were analyzed in A549, H460, and H226 cells treated with vorinostat and/or B[a]P. Western blotting was performed according to standard procedures. Antibodies against cyclin E (HE12, #4129), cyclin A2 (BF683, #4656), and cyclin B1 (#4138) were purchased from Cell Signaling Technology. (TIFF 1815 kb)

## Abbreviations

B[a]P: benzo[a]pyrene
CI: confidence interval
HDACIs: histone deacetylase inhibitors
HR: hazard ratio
NSCLC: non-small cell lung cancer
RFS: recurrence-free survival
RT-PCR: Reverse transcription polymerase chain reaction
SAHA: suberoylanilide hydroxamic acid
siRNA: small interfering RNA
TMAs: Tissue microarrays
