# Supplementary figures and images for: Negative effect of cyclin D1 overexpression on recurrence-free survival in stage II-IIIA lung adenocarcinoma and its expression modulation by vorinostat in vitro
Source: BMC Cancer. 2015 Dec 17;15:982. doi: 10.1186/s12885-015-2001-7 (PMC4683946; doi:10.1186/s12885-015-2001-7)

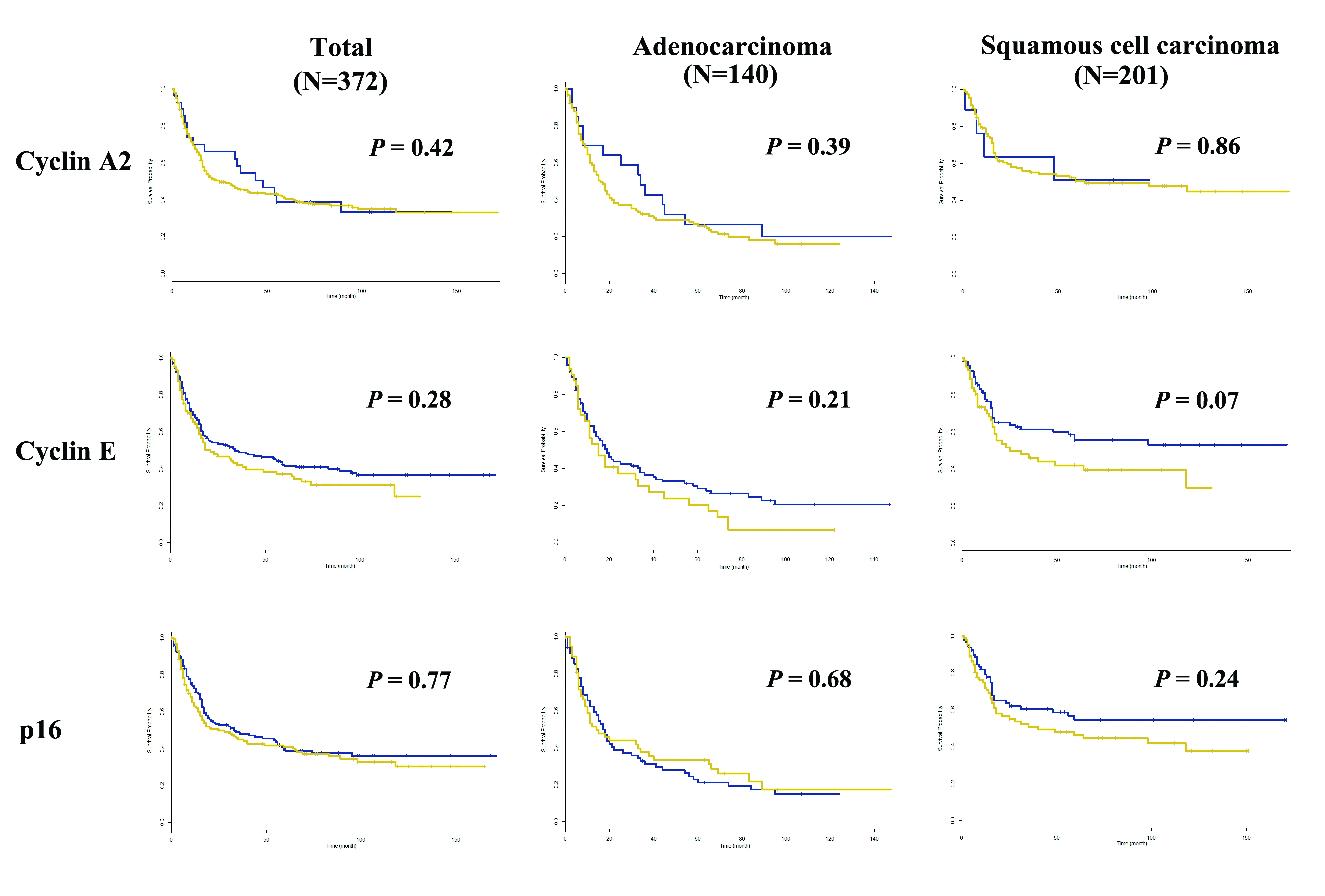

Supplement: Additional file 1: Figure S1. — Recurrence-free survival in stage II-IIIA NSCLC. Recurrence-free survival was compared according to expression statuses of cyclin A2, cyclin E, and p16. Data were stratified according to histology. The yellow and blue lines indicate groups with and without abnormal expression of each protein, respectively. P–values were based on the log-rank test. (TIFF 2996 kb) [file 12885_2015_2001_MOESM1_ESM.tiff]

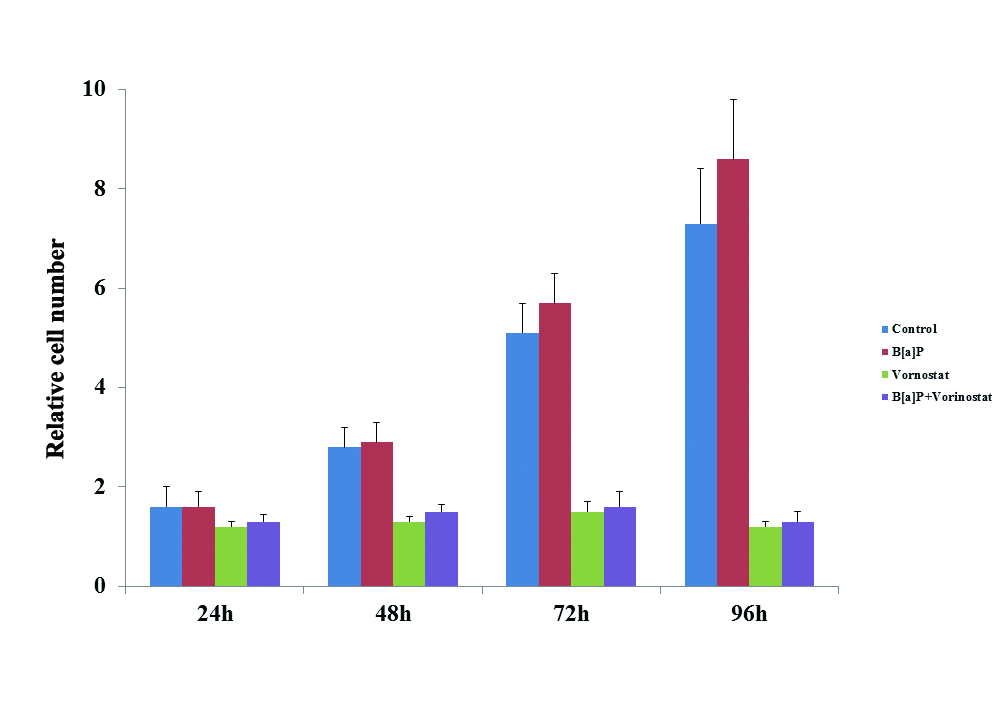

Supplement: Additional file 2: Figure S2. — Effect of low dose vorinostat on cell proliferation. A549 cells were pre-treated with 5 μM B[a]P for 9 days and then incubated in combination with 1 μM vorinostat for the indicated hours. The total number of viable cells at each time point was determined by MTT assay. The y-axis indicates cell numbers relative to time zero. Each value represents mean ± standard deviation of three experiments. (TIFF 1042 kb) [file 12885_2015_2001_MOESM2_ESM.tiff]

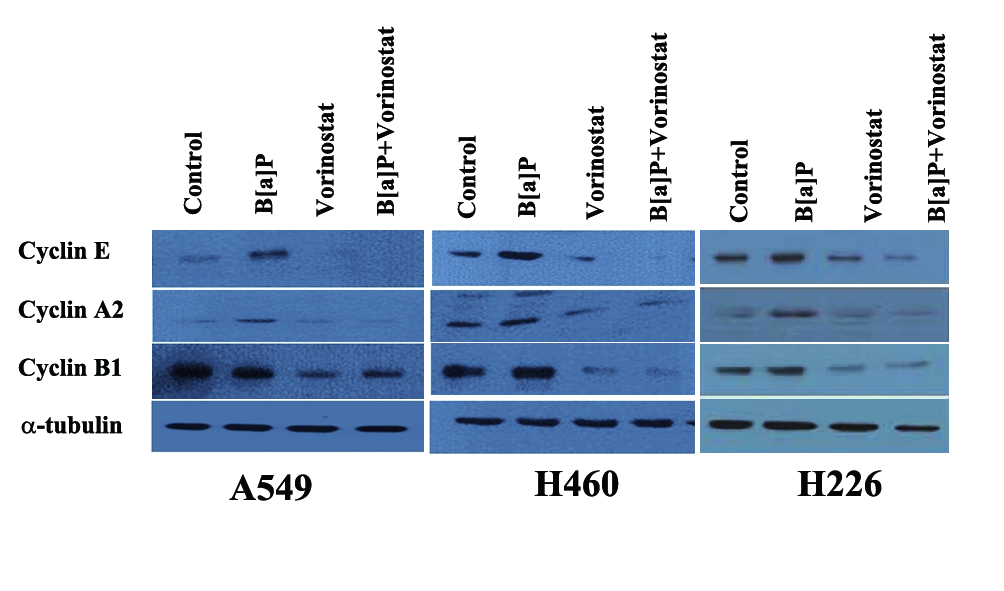

Supplement: Additional file 3: Figure S3. — The effect of vorinostat on expression of cyclins. The expression levels of cyclin E, cyclin A2, and cyclin B1 were analyzed in A549, H460, and H226 cells treated with vorinostat and/or B[a]P. Western blotting was performed according to standard procedures. Antibodies against cyclin E (HE12, #4129), cyclin A2 (BF683, #4656), and cyclin B1 (#4138) were purchased from Cell Signaling Technology. (TIFF 1815 kb) [file 12885_2015_2001_MOESM3_ESM.tiff]
